# Chemometrics of the Composition and Antioxidant Capacity of *Hyptis crenata* Essential Oils from Brazil

**DOI:** 10.3390/molecules28083371

**Published:** 2023-04-11

**Authors:** Maria Nancy N. de Lima, Jamile Silva da Costa, Bruna A. Guimarães, Jofre Jacob S. Freitas, William N. Setzer, Joyce Kelly R. da Silva, José Guilherme S. Maia, Pablo Luis B. Figueiredo

**Affiliations:** 1 Programa de Pós-Graduação em Química, Instituto de Ciências Exatas e Naturais, Universidade Federal do Pará, Belém 66075-110, Braziljoycekellys@ufpa.br (J.K.R.d.S.); gmaia@ufpa.br (J.G.S.M.); 2 Laboratório de Química dos Produtos Naturais, Universidade do Estado do Pará, Belém 66087-662, Brazil; 3 Programa de Pós-Graduação em Farmacologia e Bioquímica, Instituto de Ciências Biológicas, Universidade Federal do Pará, Belém 66075-110, Brazil; 4 Laboratório de Morfofisiológia Aplicada a Saúde, Departamento de Morfologia e Ciências Fisiológicas, Universidade do Estado do Pará, Belém 66087-662, Brazil; 5 Aromatic Plant Research Center, 230 N 1200 E, Suite 100, Lehi, UT 84043, USA; wsetzer@chemistry.uah.edu; 6 Programa de Pós-Graduação em Química, Universidade Federal do Maranhão, São Luís 65080-040, Brazil; 7 Programa de Pós-Graduação em Ciências Farmacêuticas, Universidade Federal do Pará, Belém 66075-110, Brazil

**Keywords:** multivariate analysis, volatiles, DPPH, monoterpenes, sesquiterpenes

## Abstract

*Hyptis crenata* (Pohl) ex Benth is used in traditional medicine as an analgesic to treat general pain. Six *Hyptis crenata* samples (Hc-1 to Hc-6) were collected in Pará state, Brazil. The leaf essential oils were obtained by hydrodistillation, and GC-MS and GC-FID were used to analyze their chemical compositions. The antioxidant capacity was measured in vitro using DPPH and carotene/linoleic acid assays. Chemometrics analysis (PCA, HCA, and clustered heat map) were used to identify the sample relationships between those collected in this study and those from the literature (Hc-7 to Hc-16) samples. According to the main chemical constituents identified in the samples described in this work and the literature, the sixteen samples were classified into ten groups. Group I was characterized by 1,8-cineole (31.0%), α-pinene (13.6%), (*E*)-caryophyllene (7.8%), and β-pinene (7.6%); and Group IV was characterized by 1,8-cineole (17.4–23.5%), α-pinene (15.7–23.5%), β-pinene (10.5–13.4%), and limonene (8.5–9.7%). Both groups are described for the first time. The total antioxidant capacity was expressed in Trolox Equivalent Antioxidant Capacity values (TEAC): TEAC of Hc-5 (551.9 mg.TE/g) and Hc-6 (475.1 mg.TE/g). In the β-carotene/linoleic acid assay, the highest inhibition was from Hc-2 (40.0%), Hc-6 (39.0%), and Hc-3 (29.4%).

## 1. Introduction

Lamiaceae comprises over 200 genera and 7000 species as the sixth largest family of angiosperms. Its species have economic, medicinal, and ecological importance [1]. *Hyptis* Jacq. is the largest genus of the subtribe Hyptidinae, which includes 19 genera, with 144 species occurring mainly in tropical America [2]. *Hyptis* species have constituents with pharmacological potential, with antibacterial, antifungal, anti-inflammatory, antioxidant, and cytotoxic properties [3].

**Figure 1:**
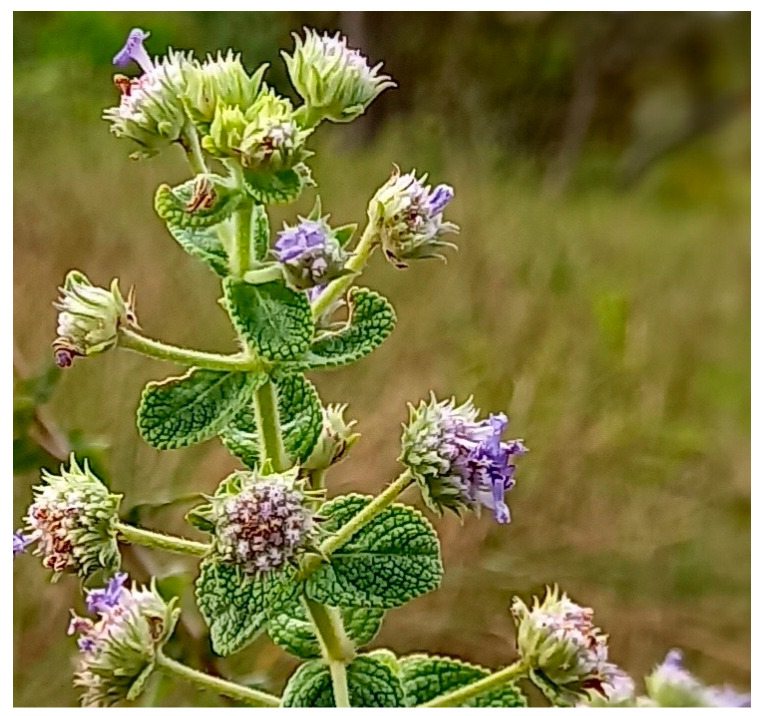
*Hyptis crenata* (Pohl) ex Benth.

*Hyptis crenata* (Pohl) ex Benth (syn. *Hyptis crenata* var. *hirsuta* Pohl ex J.A.Schmidt, *Hyptis crenata* var. *microphylla* Pohl ex J.A.Schmidt, *Mesosphaerum crenatum* Kuntze, *Mesosphaerum crenatum* var. *albiflorum* Kuntze, and *Mesosphaerum crenatum* var. *subviolacum* Kuntze) [4] is popularly known as “salva,” “salva-do-marajó,” “salsa-do-campo,” “hortelã-brava,” “hortelã-do-campo,” and “hortelãzinha” [5]. *Hyptis crenata* (Figure 1) can be shrub or subshrub with erect stems that are branched and densely villous. The leaves are spreading or sometimes slightly deflexed, sessile, membranous, rugose, ovate to ovate-oblong, apex obtuse to acute, and base rounded to subcordate. Its inflorescence appear with pedunculate capitula, most of which positions near the apex and sub-corymbose. Capitula are semiglobose, involucral bracteoles, and linear-subulate to lanceolate. Flowers have fruiting calyx tubes with lobes that are lanceolate-acuminate to subulate. It is native to Brazil, mainly distributed in the north, northeast, midwest, and southeast regions [6].

Ethnopharmacological studies carried out in the Brazilian Tropical Atlantic Forest reported that the leaf decoction of this species is used as an analgesic, and the infusion of the roots is used to treat general pains, bad cold, rheumatism, and menstrual colic [7]. In the Brazilian Pantanal region, leaf tea made from the plant is used for antiulcer and anti-inflammatory treatments [8].

The biological properties of essential oils are very likely influenced by the plant’s phytochemical composition. For example, there are six chemotypes of tea tree (*Melaleuca alternifolia* Cheel), but commercial tea tree essential oils are always the terpinen-4-ol chemotype, which is used to treat several skin conditions such as acne, eczema, herpes simplex, wounds, burns, insect bites, and mycoses [9]. The thymol/carvacrol chemotypes of *Thymus vulgaris* L. essential oils demonstrated significantly greater antioxidant activity than chemotypes with low thymol and carvacrol concentrations [10]. Similarly, the *Piper betle* L. essential oil with the highest chavibetol concentration also exhibited the best DPPH and ABTS radical-scavenging activity [11].

Likewise, the essential oil of *H. crenata* has shown chemical variability due to intraspecific variations [5]. In addition, the *H. crenata* oils exhibit bactericidal, fungicidal [12], antiulcer [13], anti-inflammatory, and antinociceptive properties [14].

Therefore, due to the pharmacological and biological potential presented by *Hyptis crenata*, this work aimed to investigate the chemical variability of *H. crenata* essential oils and their antioxidant capacity by applying chemometric analysis. We hypothesize that H. crenata will exhibit variation in volatile chemical profiles and that these variations are likely to affect biological properties such as antioxidant activities.

## 2. Results and Discussion

### 2.1. Yield and Chemical Composition of the Essential Oil

The essential oils of six *Hyptis crenata* specimens evaluated in this work showed chemical variability. The yield ranged from 1.1 to 3.1%, as shown in Table 1. The quantification and identification of 88 constituents in the analyzed oils represent an average of 97.7% of the total oil content.

The essential oils from dry and fresh aerial parts of *Hyptis crenata* sampled in Marajó Island (Brazilian Amazon) presented yields of 0.9 and 1.4%, respectively, the highest yield reported in the literature [17]. Another study that evaluated the chemical variability of essential oils from aerial parts, collected in Pará state and Tocantins (Brazilian Amazon), reported that the yield ranged from 0.2 to 0.9% [5], values lower than those of this work. Another sample from the Brazilian Cerrado exhibited an oil content of 0.6% [12]. Therefore, the yields reported in the literature (0.2–1.4%) were lower than those described in this study (1.1–3.1%).

Monoterpene hydrocarbons (29.5–56.9%) and oxygenated monoterpenes (31.2–59.0%) were predominant in the essential oils. The main compounds (>5%) identified in the essential oils were the monoterpene with terpinane (1,8-cineole, 17.0–31.5%; limonene, 0–9.7%), pinane (α-pinene, 10.5–23.5%; β-pinene, 0–13.4%), and bornane skeletons (camphor, 1.9–19.3%; borneol, 0–17.4%), followed by the sesquiterpenes with caryophyllane ((*E*)-caryophyllene, 0.9–7.8%), and longipinane skeletons (α-longipinene, 0.1–5.2%).

The oil samples were grouped into three chemical groups according to the main chemical constituents identified. Group I, corresponding to Hc-1 oil, was characterized by 1,8-cineole (31.0%), α-pinene (13.6%), (*E*)-caryophyllene (7.8%), and β-pinene (7.6%); this chemical group has not been previously described in the literature and is presented here for the first time. Group II (Hc-2) was dominated by 1,8-cineole (19.2%), camphor (17.6%), α-pinene (13.0%), and β-pinene (9.1%); this group was previously described in a sample collected in Salvaterra, Pará, Brazil (Hc-13), composed of 1,8-cineole (23.2%), α-pinene (19.5%), β-pinene (13.8%), and camphor (11.6%) [17]. Group III (Hc-3) was characterized by the contents of 1,8-cineole (31.5%), α-pinene (21.8%), and β-pinene (9.8%); a sample from Melgaço, Pará (Hc-11) also showed 1,8-cineole (36.7%), α-pinene (14.5%), and β-pinene (7.9%) as main compounds [5]. Group IV (Hc-4 and -6) was composed of 1,8-cineole (17.4–23.5%), α-pinene (15.7–23.5%), β-pinene (10.5–13.4%), and limonene (8.5–9.7%); the Hc-10 and -12 oils, extracted from specimens collected in the municipalities of Marajó, Pará state, were also composed of α-pinene (22.0–51.1%), 1,8-cineole (16.5–17.6%), β-pinene (10.3–17.0%), and limonene (5.4–15.0%) [5,17]. Group V (Hc-5), characterized by camphor (19.3%), 1,8-cineol (18.4%), and borneol (16.4%), is described for the first time. Group VI (Hc-7) occurred in a sample from Mato Grosso do Sul state characterized by camphor (17.3%), α-pinene (15.5%), (*E*)-caryophyllene (10.7%), and β-pinene (10.5%) [18]. Group VII (Hc-8) from Tocantins state (Brazil), was rich in terpinolene (37.8%), (*E*)-caryophyllene (9.9%), limonene (6.4%), and α-pinene (6.1%) [5]. Group VIII (Hc-9), sampled in São Sebastião da Boa Vista (Pará state, Brazil), was rich in 1,8-cineole (23.9%), borneol (21.8%), and (*E*)-caryophyllene (18.8%) [5]. Group IX (Hc-14) was collected in the Mato Grosso state and was characterized by borneol (17.8%), 1,8-cineole (15.6%), and *p*-cymene (7.9%) [12]. Group X (Hc-15 and -16), collected in Maranhão state (Brazil), was rich in camphor (32.8–33.7%), 1,8-cineole (18.0–19.8%), α-pinene (13.4–15.2%), (*E*)-caryophyllene (8.0–13.0%), and *p*-cymene (5.4–6.9%) [13,19].

Therefore, according to the main chemical constituents identified in the samples described in this work (Hc-1 to -6) and the literature (Hc-7 to -16), the sixteen samples were classified in ten chemical groups. Two chemical groups (group I and V) are described for the first time.

### 2.2. Multivariate Analyses of Hyptis crenata Specimens

The chemical variability of *Hyptis crenata* oil samples was evaluated by multivariate statistical analyses (PCA, Principal Components Analysis; HCA, Hierarchical Cluster Analysis). The total percentage of monoterpene hydrocarbons (MH), oxygenated monoterpenes (OM), sesquiterpene hydrocarbons (SH), oxygenated sesquiterpenes (OS), and other compounds (OT) were obtained from oil samples, according to the original citations (Table 1 and Table A1). The data were used as variables (see Table A2, Appendix B).

**Figure 2:**
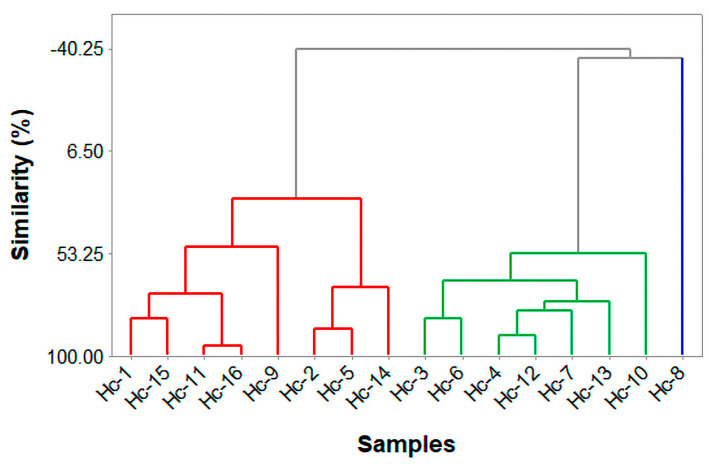
Hierarchical cluster analysis of *Hyptis crenata* essential oils samples.

The HCA (Figure 2) shows the formation of three groups I (Chemotypes). The first one comprises eight samples:Hc-1, -2, -5, -9, -11, -14, -15, and -16. The second group comprises Hc-3, -4, -6, -7, -10, -12, and -13 samples. The third group comprises the Hc-8 sample.

**Figure 3:**
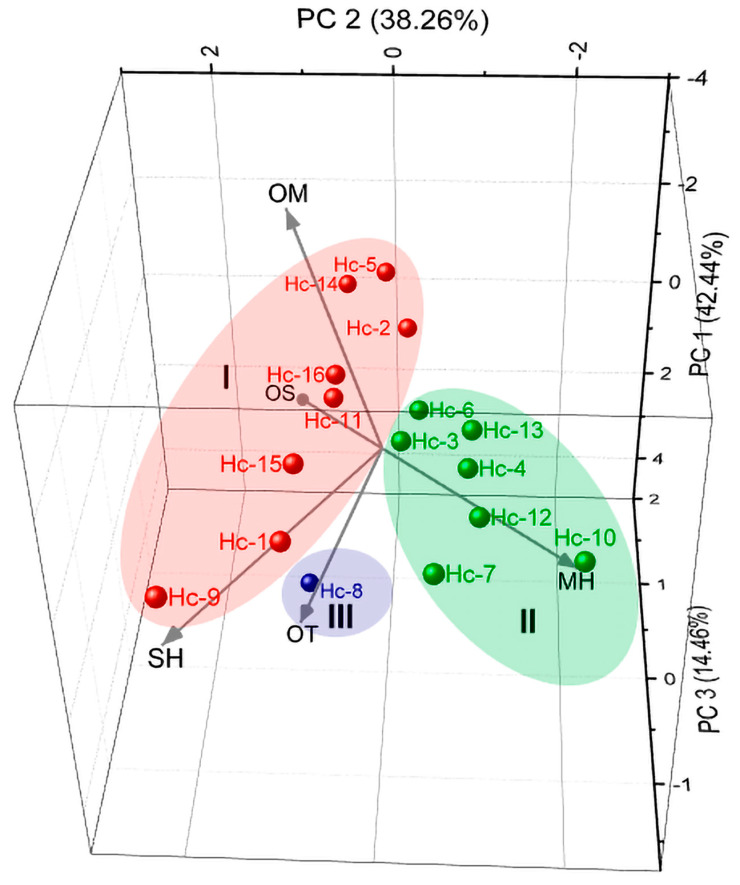
Principal Component Analysis of *Hyptis crenata* oil samples.

The Principal Components Analysis (PCA, Figure 3) elucidated 95.2% of the data variability. PC1 explained 42.4% and showed positive correlations with the monoterpene hydrocarbons (MH, r = 0.34), sesquiterpene hydrocarbons (SH, r = 0,12), oxygenated sesquiterpenes (OS, r = 0.41), and other compounds (OT, r = 0.60), as well as negative correlations with oxygenated monoterpenes (OM, r = −0.59). The second component explained 38.3% and presented positive correlations with the oxygenated monoterpenes (OM, r = 0.29), sesquiterpene hydrocarbons (SH, r = 0.59), oxygenated sesquiterpenes (OS, r = 0.35), and other compounds (OT, r = 0.27), as well as a negative correlation with monoterpene hydrocarbons (MH, r = −0.61). The third component, PC3, explained 14.5% of the data and displayed positive correlations with the oxygenated monoterpenes (OM, r = 0.26) and oxygenated sesquiterpenes (r = 0.73), as well as negative correlations with monoterpene hydrocarbons (MH, r = −0.09), sesquiterpene hydrocarbons (SH, r = −0.63), and other compounds (OT, r = −0.06). Like HCA, the PCA analysis confirmed the formation of three distinct groups (Chemotypes).

Chemotype I was characterized by the highest amounts of oxygenated monoterpenes (38.7–59.0%), followed by monoterpene hydrocarbons (10.2–41.8%), and minor amounts of sesquiterpene hydrocarbons (2.0–29.4%), oxygenated sesquiterpenes (1.5–6.8%), and other compounds (0–1.5%). Chemotype II was characterized by the highest amounts of monoterpene hydrocarbons (44.8–80.4%), followed by oxygenated monoterpenes (18.1–42.9%), and minor amounts of sesquiterpene hydrocarbons (1.2–10.7%), oxygenated sesquiterpenes (0–5.0%), and other compounds (0–0.1%). Chemotype III was characterized by the highest amounts of monoterpene hydrocarbons (58.7%), sesquiterpene hydrocarbons (20.0%), minor amounts of oxygenated sesquiterpenes (9.8%), other compounds (5.3%), and oxygenated monoterpenes (1.6%).

**Figure 4:**
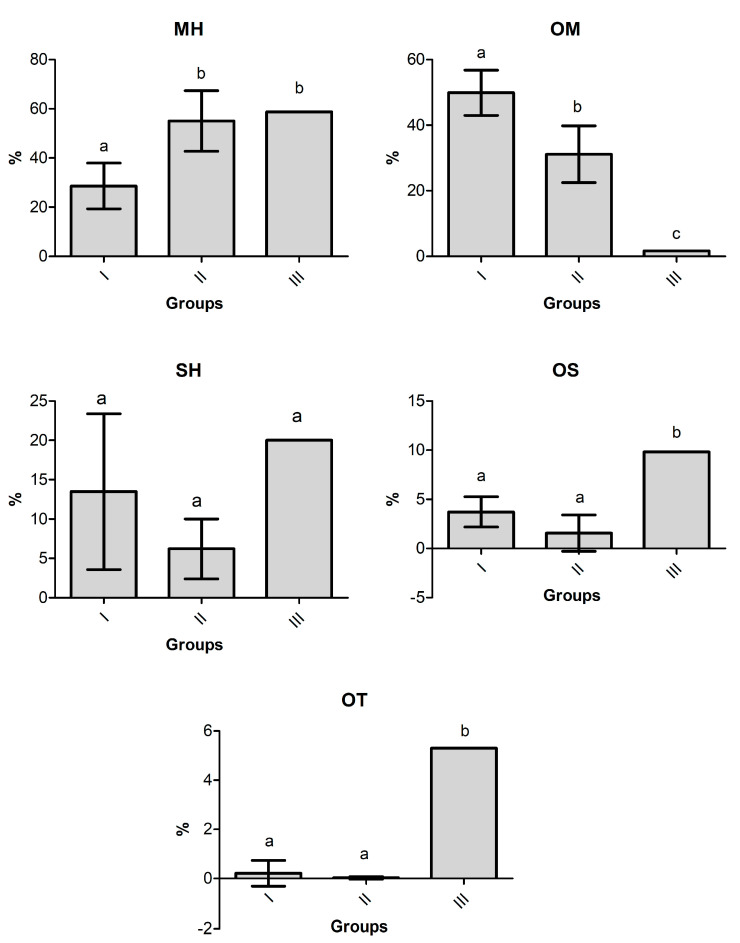
Compound classes of the *H. crenata* chemotypes. Mean ± standard deviation. Values with the same letters (a or b) in the bars do not differ statistically in the Tukey test (*p* > 0.05). MH—monoterpene hydrocarbons, OM—oxygenated monoterpenes, SH—sesquiterpene hydrocarbons, OS—oxygenated sesquiterpenes, OT—other compounds.

Analyzing the mean contents and standard deviations of the compound classes present in *Hyptis crenata* oil chemotypes (Figure 4) showed that Group I was statistically different (Tukey test, *p* < 0.05) from Group II by the content of monoterpene hydrocarbons (I = 28.6 ± 9.3%; II = 55.0 ± 12.3%) and oxygenated monoterpenes (I = 49.9 ± 6.9%; II = 31.1 ± 8.6%). Furthermore, Group III was distinguished from the other groups by the content of oxygenated monoterpenes (1.6 ± 0.0%), oxygenated sesquiterpenes (9.8 ± 0.0%), and other compounds (5.3 ± 0.0%).

**Figure 5:**
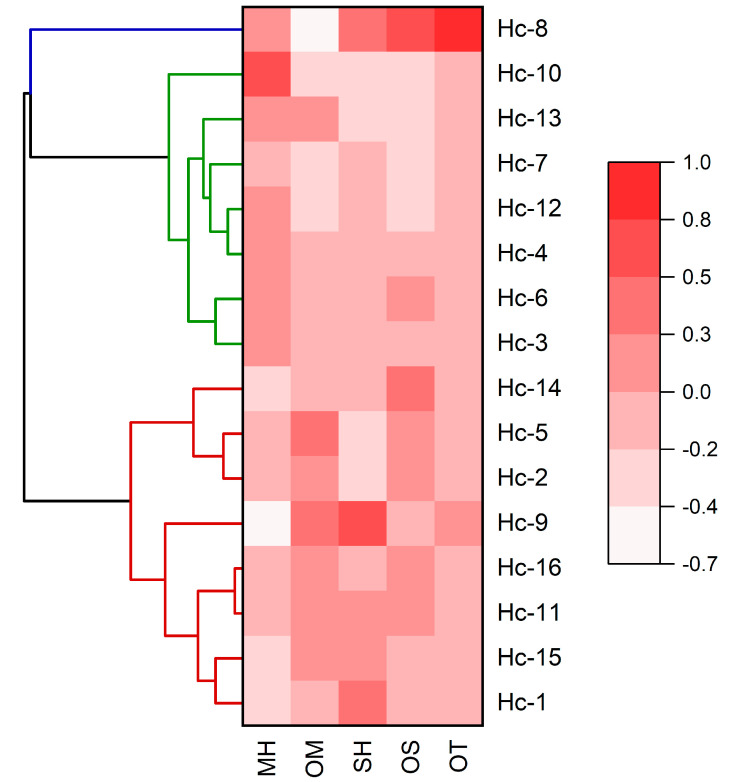
Clustered heat map of the volatile classes of *Hyptis crenata* samples.

Applying additional multivariate analyses in the heatmap analysis combined with hierarchical clustering analysis with the compound classes, the color pattern varied with color intensity and increased gradually, from lowest to the highest grade. The clustered heatmap (Figure 5) confirmed the above clustering results for HCA and PCA.

Several studies have suggested variation in essential oil compositions may be due to climatic [20,21,22], edaphic [23], altitudinal [24,25], genetic [26,27,28], or phenological [29,30] factors.

### 2.3. Antioxidant Activity

**Figure 6:**
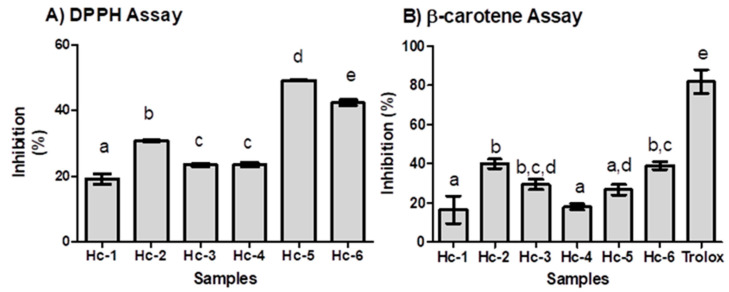
Antioxidant activity of essential oils of *Hyptis crenata:* (**A**) DPPH Assay (**B**) β-carotene assay. Values with the same letters in the bars (a, b, c, d or e) do not differ statistically in the Tukey test (*p* > 0.05).

The antioxidant activity of the oil samples was evaluated in two different systems (DPPH radical-scavenging and β-carotene/linoleic acid assays). The samples were effective in the DPPH (19.2–49.3%) and β-carotene/linoleic (16.4–40.0%) assays (Table A3, Appendix C and Figure 6).

The greater inhibition rate of DPPH was observed in the oil samples Hc-5 (49.3%) and Hc-6 (42.4%); both samples showed higher amounts of 1,8-cineole (Hc-5: 18.4%, Hc-6: 17.4%) and α-pinene (Hc-5: 10.5%, Hc-6: 15.7%). However, the sample Hc-5 e Hc-6 presented great differences in amounts of camphor (Hc-5: 19.3%; Hc-6: 4.5%), borneol (Hc-5: 16.4%; Hc-6: 2.6%), and β-pinene (Hc-5: 7.5%; Hc-6: 13.4%). 

The total antioxidant capacity was expressed in Trolox Equivalent Antioxidant Capacity values (TEAC, mg.TE/g). The TEAC values of Hc-5 (551.9 mg.TE/g) and Hc-6 (475.1 mg.TE/g) were about half the values of Trolox. On the other hand, lower inhibition was observed in the oil samples Hc-1 (19.2 ± 1.6%), Hc-3 (23.5 ± 0.5), and Hc-4 (23.5 ± 0.7%). The high amounts of 1,8-cineole, and α-pinene characterized these samples. Sharopova et al. [31] and Choi et al. [32] have reported that the monoterpene β-Pinene (IC_50_ 3116.3 μg/mL) shows weak antioxidant capacity in the DPPH method. Moreover, 1,8-cineole displayed DPPH radical scavenging with an IC_50_ of 912.9 μg/mL [31].

In the β-carotene/linoleic acid assay, the highest inhibition was from Hc-2 (40.0%), Hc-3 (29.4%), and Hc-6 (39.0%), only about one-third the values of Trolox, followed by Hc-5 (26.7%), Hc-4 (18.0%), and Hc-1 (16.4%).

Nonpolar antioxidants exhibit stronger antioxidative properties in emulsions because they are concentrated in the lipid phase, thus, ensuring high protection to the emulsion. On the other hand, polar antioxidants remaining in the aqueous phase are more diluted and, therefore, less effective in protecting the lipid phase [33]. Moreover, the DPPH assay is performed in a polar system, and the β-carotene/linoleic acid assay is performed in an apolar system [34].

Rebelo et al. [17] performed the DPPH assay on essential oil samples from fresh leaves and methanolic extract of *H. crenata* at different concentrations. The inhibition values obtained were 42.6–79.9%, 24.5–71.4%, and 14.2–94.0%, respectively. In another assay, using the ABTS method, the inhibition of leaves and flowers of *H. crenata* at different extraction times was 26.0–65.8% for the leaves and 55.6–84.4% for the flowers [35].

## 3. Materials and Methods

### 3.1. Plant Material

The leaves of the six *Hyptis crenata* specimens were collected in Marajó Island, Pará state, Brazil, during the rainy season (August-December). The collection site, herbarium voucher number, and geographic coordinates are listed in Table 2. The plant specimens were deposited in the Herbarium of Museu Paraense Emílio Goeldi (MG) in the city of Belém, Brazil.

The leaves were dried for three days at room temperature, then pulverized. The leaves were submitted to essential oil hydrodistillation in duplicate using a Clevenger-type apparatus (2 h). The oils obtained were dried over anhydrous sodium sulfate, and total oil yields were expressed as mL/100 g of the dried material [36].

### 3.2. Analysis of Essential Oil Composition

GC-MS and GC-FID were performed to analyze the oil composition. A Shimadzu instrument Model QP 2010 ultra (Shimadzu, Tokyo, Japan) was used. An Rtx-5MS (30 m × 0.25 mm; 0.25 μm film thickness) fused silica capillary column (Restek, Bellefonte, PA, USA) was used as stationary phase. Helium was the carrier gas adjusted to 1.0 mL/min at 57.5 kPa with a split injection mode (split ratio 1:20) of 1 μL of *n*-hexane solution (oil 5 μL: 500 μL *n*-hexane); injector and interface temperature were 250 °C; oven programmed temperature was 60 to 240 °C (3 °C/min), followed by an isotherm of 10 min. With EIMS (Electron Ionization Mass Spectrometry) at 70 eV, the ion source temperature was 200 °C.

The mass spectra were obtained by automatic scanning every 0.3 s, with mass fragments in the range of 35–400 *m*/*z*. The compounds present in the samples were identified by comparison of their mass spectra and retention indices, calculated for all volatile components using a linear equation of Van Den Dool and Kratz [37], with the data present in the commercial libraries FFNSC-2 [16] and Adams [15]. The retention index was calculated using *n*-alkane standard solutions (C8–C40, Sigma-Aldrich, St. Louis, MO, USA) in the same chromatographic conditions.

The GC-FID analysis was carried out on a Shimadzu QP-2010 instrument (Shimadzu, Tokyo, Japan), equipped with an FID detector, in the same conditions, except that hydrogen was used as the carrier gas. The percentage composition of the oil samples was computed from the GC-FID peak areas. The analyses were carried out in triplicate.

### 3.3. DPPH Radical Scavenging Assay

The antioxidant activity of the oils samples was evaluated by the DPPH radical scavenging method as described by Figueiredo et al. [33]. The 2,2-Diphenyl-1-picrylhydrazyl (DPPH) is a stable dark-violet free radical with maximum absorption at 517 nm, which is reduced in the presence of antioxidants.

The DPPH was diluted to initial absorbance of 0.62 ± 0.02 at 517 nm and room temperature. Each essential oil sample (50 μL, 10 mg/mL) was mixed with Tween 20 solution (0.5%, 50 μL, *w*/*w*) and then added to DPPH (0.5 mM, 1900 μL) in ethanol. For each sample, an ethanol blank was also measured. The absorbance was measured at the start of the reaction (time zero), each 5 min during the first 30 min, and then at continuous intervals of 30 min up to constant absorbance (plateau of reaction, 2 h) in Ultrospec™ 7000 spectrophotometer (Biochrom US, Holliston, MA, USA). The standard curves were prepared using Trolox (6-hydroxy-2,5,7,8-tetramethylchroman-2-carboxylic acid) (Sigma-Aldrich, St. Louis, MO, USA) at concentrations of 60, 120, 240, and 480 μg/mL. The results were expressed as milligrams of Trolox (mg TE/g) equivalents per gram of the sample.

### 3.4. β-Carotene/linoleic Acid Assay

The amount of 0.2 mg of β-carotene was dissolved in 1 mL of chloroform (HPLC grade), and 25 μL of linoleic acid and 200 μL of Tween 20 were added. Chloroform was wholly evaporated using a vacuum evaporator. Then, 50 mL of oxygen-saturated water was added with vigorous shaking. An aliquot of 2300 μL of this reaction mixture was dispensed into test tubes, and 200 μL portions of the oil samples (1.0 mg/mL in ethanol) were added. This emulsion system was incubated at 50 °C. The same procedure was repeated with Trolox and a blank of ethanol.

The absorbance of these solutions was recorded at 470 nm and monitored at intervals of 15 min, for 120 min in Ultrospec™ 7000 spectrophotometer (Biochrom US, Holliston, MA, USA). The antioxidant activity (AA%) was calculated regarding the percent inhibition relative to the control using the equation AA% = [1 − (Abssample − Abssample)/(Abscontrol − Abscontrol)] × 100. All experiments were triplicated [33].

### 3.5. Multivariate Statistical Analysis

The multivariate statistical analysis was carried out to discern any relationship among *Hyptis crenata* oil samples (described in Appendix A). The total percentage of the monoterpene hydrocarbons (MH), oxygenated monoterpenes (OM), sesquiterpene hydrocarbons (SH), and oxygenated sesquiterpenes (OS) of each oil was extracted from the literature (Table A1). The 17 × 6 data matrix was used for variables (see Appendix B). The matrix was standardized for the multivariate analysis by subtracting the mean and then dividing it by the standard deviation.

Hierarchical grouping analyses (HCA) were performed considering the Euclidean distance and the Ward linkage (Minitab free 390 version, Minitab Inc., State College, PA, USA). The principal component analysis (PCA) was applied to verify the interrelation (OriginPro trial version, OriginLab Corporation, Northampton, MA, USA) [38]. A clustered heat map was constructed using Euclidean distance via the Ward linkage (OriginPro trial version, OriginLab Corporation, Northampton, MA, USA).

The antioxidant capacity was calculated in triplicate, and the data were expressed as mean± SD. Statistical differences were evaluated by Tukey’s test (*p* < 0.05) using the software GraphPad Prism 6.0.

## 4. Conclusions

The present study showed two new chemotypes of *Hyptis crenata* essential oil. The first one is rich in 1,8-cineole, α-pinene, (*E*)-caryophyllene, and β-pinene; and the second is rich in 1,8-cineole, α-pinene, β-pinene, and limonene. The essential oils displayed differences in their antioxidant activity.

Further research on essential oils from other plant parts (stems, roots, flowers) should also be explored in future study. Since there is intraspecific chemical variability in *H. crenata*, prior chemical knowledge must be available before suggesting its use for phytomedicinal purposes.

## Figures and Tables

**Table 1:** Yield and composition of essential oils from *Hyptis crenata* samples.

RI_C_	RI_L_	Constituents	Hc-1	Hc-2	Hc-3	Hc-4	Hc-5	Hc-6
					Samples (%) *			
923	921 ^a^	tricyclene	0.1	0.1		0.1	0.1	0.1
926	924 ^a^	α-thujene	0.0	0.4	0.1	0.1	0.3	0.2
**934**	**932 ^a^**	**α-pinene**	**13.6**	**13.0**	**21.8**	**23.5**	**10.5**	**15.7**
947	945 ^a^	α-fenchene	0.3	0.2	0.7	0.8		0.7
949	946 ^a^	camphene	1.7	2.9	3.3	3.8	3.1	3.1
954	953 ^a^	thuja-2,4(10)-diene	tr		0.1	tr		tr
973	969 ^a^	sabinene		0.2	0.1	0.1	0.2	0.1
**978**	**974 ^a^**	**β-pinene**	**7.6**	**9.1**	**9.8**	**10.5**	**7.5**	**13.4**
982	973 ^a^	*trans*-*p*-menthane				0.1		
991	988 ^a^	myrcene	0.9	1.6	2.4	2.2	1.3	2.3
1006	1002 ^a^	α-phellandrene	0.3	0.4	0.8	0.7	0.3	0.8
1011	1008 ^a^	δ-3-carene		0.2	0.7			
1017	1014 ^a^	α-terpinene	0.6	1.0	1.1	1.0	0.8	1.3
1024	1089 ^a^	*p*-cymene	0.8	2.9	1.7	2.0	2.2	2.0
**1030**	**1024 ^a^**	**limonene**	**2.0**	**5.1**		**9.7**	**4.5**	**8.5**
**1033**	**1026 ^a^**	**1,8-cineole**	**31.0**	**19.2**	**31.5**	**23.5**	**18.4**	**17.4**
1058	1054 ^a^	γ-terpinene	1.0	3.1	1.4	1.2	2.4	2.1
1066	1065 ^a^	*cis*-sabinene hydrate (IPP vs. OH)		0.2	0.1		0.2	0.1
1099	1098 ^a^	*trans*-sabinene hydrate					0.3	
1071	1067 ^a^	*cis*-linalool oxide (furanoid)		0.1				
1089	1086 ^a^	terpinolene	0.5	1.5	1.4	1.0	1.2	1.4
1100	1095 ^a^	linalool	0.1	0.5			0.2	
1114	1114 ^a^	*endo*-fenchol	0.2	0.2	0.3	0.3	0.1	0.4
1121	1119 ^a^	*trans*-pinene hydrate	0.1		0.2		0.1	
1121	1118 ^a^	*cis*-*p*-menth-2-en-1-ol		0.2				0.2
1139	1136 ^a^	*trans*-*p*-menth-2-en-1-ol	tr	0.1	0.1			
**1144**	**1141 ^a^**	**camphor**	**1.9**	**17.6**	**2.4**	**3.8**	**19.3**	**4.5**
1148	1145 ^a^	camphene hydrate	0.7	0.5	0.7	0.5	0.3	0.7
1157	1155 ^a^	isoborneol	1.4	0.1	tr		0.1	
1162	1160 ^a^	pinocarvone	tr	0.1	0.1	0.2		0.2
**1166**	**1165 ^a^**	**borneol**		**6.7**	**1.6**	**1.8**	**16.4**	**2.3**
1177	1174 ^a^	terpinen-4-ol	0.5	1.3	0.8	0.8	1.1	1.2
1185	1179 ^a^	*p*-cymen-8-ol	tr	0.1			0.1	
1191	1186 ^a^	α-terpineol	2.6	2.3	1.8	2.5	1.8	3.9
1197	1194 ^a^	myrtenol	0.1	0.3	0.4	0.4	0.2	0.5
1295	1289 ^a^	thymol	tr	0.3	0.1		0.2	
1295	1297 ^a^	carvacrol ethyl ether		0.1			0.1	
1302	1298 ^a^	carvacrol	tr	0.1			0.1	
**1352**	**1350 ^a^**	**α-longipinene**	**5.2**	**0.2**	**1.0**	**0.9**	**0.1**	**1.0**
1357	1356 ^a^	eugenol		0.1	0.1	0.1	tr	
1372	1373 ^a^	α-ylangene	0.1		0.1			
1374	1374 ^a^	isoledene	tr	tr	0.1			
1377	1374 ^a^	α-copaene	0.1		0.1			
1400	1407 ^a^	longifolene	0.1					
1411	1409 ^a^	α-gurjunene		tr	0.1			
**1421**	**1417 ^a^**	**(*E*)-caryophyllene**	**7.8**	**1.2**	**2.9**	**2.0**	**0.9**	**4.3**
1429	1439 ^a^	aromadendrene	1.1	1.0	1.1	0.9	0.6	1.4
1429	1430 ^b^	γ-maaliene		0.1	0.1	0.1	tr	0.1
1435	1436 ^b^	α-maaliene	0.1	0.1	0.1	0.1	tr	
1444	1445 ^a^	myltayl-4(12)-ene			0.2			
1444	1545 ^b^	selina-5,11-diene		0.1		0.1		0.2
1450	1449 ^a^	α-himachalene	1.0		0.6	0.1		0.2
1454	1452 ^a^	α-humulene	0.4	0.1	0.2	0.1		0.3
1462	1464 ^a^	9-*epi*-(*E*)-caryophyllene	0.1	0.2	0.2	0.1	0.1	
1473	1475 ^a^	γ-gurjunene	0.1		0.1			
1479	1481 ^a^	γ-himachalene	1.4		0.8	0.2		0.2
1482	1485 ^a^	11α-himachala-1,4-diene	0.5		0.1			
1487	1489 ^a^	β-selinene	tr		0.1			
1496	1496 ^a^	viridiflorene	0.5	0.4	0.5	0.3	0.2	
1502	1500 ^a^	β-himachalene	3.7	0.1	0.8	0.5		0.6
1508	1511 ^a^	δ-amorphene	0.1					
1514	1516 ^a^	α-dehydro-*ar*-himachalene	0.4					
1515	1513 ^a^	γ-cadinene			0.2	0.1		0.2
1524	1522 ^a^	δ-cadinene	0.2		0.2			0.1
1529	1530 ^a^	γ-dehydro-*ar*-himachalene	0.3		tr			
1536	1540 ^b^	selina-4(15),7(11)-diene	tr		0.1			
1539	1545 ^a^	selina-3,7(11)-diene			0.1			
1543	1544 ^a^	α-calacorene	0.4					
1560	1562 ^a^	*epi*-longipinanol	0.1					
1567	1566 ^a^	maaliol	0.1	tr	0.1			
1570	1570 ^a^	caryophyllenyl alcohol	0.1					
1578	1577 ^a^	spathulenol	0.1	0.4	0.2	0.2	0.2	0.3
1584	1585 ^a^	caryophyllene oxide	0.6	1.2		0.8	0.7	2.4
1584	1590 ^a^	globulol	0.3		0.8			0.9
1592	1592 ^a^	viridiflorol	0.1	1.0	0.7	0.1	2.1	0.6
1597	1599 ^a^	longiborneol	0.1					
1602	1600 ^a^	rosifoliol	0.1	0.1	0.1			0.1
1613	1615 ^a^	β-himachalene oxide	0.1		0.1	0.1		
1616	1618 ^a^	1,10-di-*epi*-cubenol			0.1			0.1
1620	1622 ^a^	10-*epi*-γ-eudesmol		0.5			0.7	
1620	1618 ^a^	junenol			0.1			
1637	1639 ^a^	caryophylla-4(12),8(13)-dien-5β-ol	0.4	0.1	0.1	0.1	0.1	0.5
1645	1640 ^a^	hinesol		0.3			0.2	
1646	1652 ^a^	himachalol			0.6	0.5		
1653	1656 ^a^	valerianol		0.2			0.3	
1658	1668 ^a^	14-hydroxy-9-*epi*-(*E*)-caryophyllene	1.1					
1662	1661 ^a^	allohimachalol	0.1		0.1			
1675	1675 ^a^	cadalene	0.1					
Monoterpene hydrocarbons			29.5	41.8	45.3	56.9	34.3	51.8
Oxygenated monoterpenes			38.7	49.8	40.1	33.7	59.0	31.2
Sesquiterpene hydrocarbons			23.8	3.3	9.7	5.4	2.0	8.5
Oxygenated sesquiterpenes			3.2	3.9	2.8	1.7	4.3	5.0
Other compounds			tr	0.1	0.1	0.1	tr	tr
Total (%)			95.1	98.9	97.9	97.8	99.7	96.5
Oil yield (%, *v/w*)			1.6	2.8	1.9	1.7	1.1	3.1

**RI_C_** = calculated retention index using an n-alkane standard solution (C_8_–C_40_) in Rtx-5MS column; **RI_L_** = literature retention index; Main constituents in bold, *n* = 2 (standard deviation was less than 2.0); **tr** = traces (% < 0.1); * = The percentage composition of the oil samples was computed from the GC-FID peak areas; **^a^** = Adams library [15]; **^b^** = FFNCS library [16].

**Table 2:** Collection site, herbarium voucher number, and geographic coordinates for the *Hyptis crenata* specimens.

Code	Collection Site	Voucher Number	Coordinates Latitude/Longitude
Hc-1	Salvaterra, Marajó, Pará state, Brazil	MG243648	1°51′43.71″ S/48°37′23.33″ W
Hc-2	Cachoeira do Arari, Marajó, Pará state, Brazil	MG238838	0°54′27.77″ S/48°40′30.45″ W
Hc-3	Salvaterra, Marajó, Pará state, Brazil	MG246271	0°52′7.04″ S/48°37′38.06″ W
Hc-4	Salvaterra, Marajó, Pará state, Brazil	MG246272	0°51′52.72″ S/48°37′9.69″ W
Hc-5	Salvaterra, Marajó, Pará state, Brazil	MG238839	0°51′42.71″ S/48°37′23.87″ W
Hc-6	Cachoeira do Arari, Marajó, Pará state, Brazil	MG238843	0°54′27.74″ S/48°40′3.51″ W

## Data Availability

The data presented in this study are available on request from the corresponding author.

## Appendix A

**Table A1:** Essential oil compositions of *Hyptis crenata*.

Sample Code	Occurrence	Plant Part/ Extraction Type	Primary Components (>5%)	Oil Yield (%)	Ref.
Hc-7	Mato Grosso do Sul, Brazil	Fresh material (HD)	camphor (17.3%), α-pinene (15.5%), (*E*)-caryophyllene (10.7%), β-pinene (10.5%), 1,8-cineol (8.7%), limonene (6.3%)	-	[18]
Hc-8	Porto Nacional, Tocantins, Brazil	Dried aerial parts (HD)	terpinolene (37.8%), (*E*)-caryophyllene (9.9%), limonene (6.4%), α-pinene (6.1%)	0.2	[5]
Hc-9	São Sebastião da Boa Vista, Pará, Brazil	Dried aerial parts (HD)	1,8-cineole (23.9%), borneol (21.8%), (*E*)-caryophyllene (18.8%)	0.9	[5]
Hc-10	Melgaço, Pará, Brazil	Dried aerial parts (HD)	α-pinene (51.1%), 1,8-cineole (16.5%) limonene (15.0), β-pinene (10.3%)	0.9	[5]
Hc-11	Melgaço, Pará, Brazil	Dried aerial parts (HD)	1,8-cineole (36.7%), α-pinene (14.5%), β-pinene (7.9%), α-terpineol (5.2%)	0.6	[5]
Hc-12	Salvaterra, Pará, Brazil	Fresh aerial parts (HD)	α-pinene (22.0%), 1,8-cineole (17.6%), β-pinene (17.0%), limonene (5.4%)	1.4	[17]
Hc-13	Salvaterra, Pará, Brazil	Dried aerial parts (HD)	1,8-cineole (23.2%), α -pinene (19.5%), β-pinene (13.8%), camphor (11.6%), borneol (5.3%)	0.9	[17]
Hc-14	Cuiabá, Mato Grosso, Brazil	Fresh aerial parts (HD)	borneol (17.8%), 1,8-cineole (15.6%), *p*-cymene (7.9%), γ-terpinene (5.3%)	0.6	[12]
Hc-15	São Raimundo das Mangabeiras, Maranhão, Brazil	Fresh aerial parts (SD)	camphor (32.8%)1,8-cineole (18.0%), α-pinene (13.4%), (*E*)-caryophyllene (13.0%), *p*-cymene (5.4%)	-	[13]
Hc-16	São Raimundo das Mangabeiras, Maranhão, Brazil	Fresh aerial parts (SD)	camphor (33.7), 1.8-cineole (19.8%), α-pinene (15.2%), and (*E*)-caryophyllene (8.0%), *p*-cymene (6.9),	-	[19]

## Appendix B

**Table A2:** Compound classes used in the multivariate statistical analyses of *Hyptis crenata* oils samples.

Samples	MH	MO	SH	OS	OT	Reference
Hc-1	29.5	38.7	23.8	3.2	0.0	*
Hc-2	41.8	49.8	3.3	3.9	0.1	*
Hc-3	45.3	40.1	9.7	2.8	0.1	*
Hc-4	56.9	33.7	5.4	1.7	0.1	*
Hc-5	34.3	59.0	2.0	4.3	0.0	*
Hc-6	51.8	31.2	8.5	5.0	0.0	*
Hc-7	44.8	26.6	10.7	0.0	0.0	[18]
Hc-8	58.7	1.6	20.0	9.8	5.3	[5]
Hc-9	10.2	55.2	29.4	1.5	1.5	[5]
Hc-10	80.4	18.1	1.4	0.0	0.0	[5]
Hc-11	32.4	49.9	13.2	3.8	0.1	[5]
Hc-12	57.8	25.4	6.6	1.3	0.0	[17]
Hc-13	48.3	42.9	1.2	0.1	0.0	[17]
Hc-14	22.6	40.9	5.5	6.8	0.0	[12]
Hc-15	26.8	52.0	18.7	2.5	0.0	[13]
Hc-16	31.0	53.4	11.9	3.7	0.0	[19]

* Data showed in Table 2.

## Appendix C

**Table A3:** Antioxidant capacity of the oils of *Hyptis crenata*.

Sample	DPPH Assay		β-Carotene Assay
	Inhibition (%) *	TEAC (mg.TE/g) *	Inhibition (%) *
Hc-1	19.2 ± 1.6 ^a^	214.8 ± 17.6 ^a^	16.4 ± 7.0 ^a^
Hc-2	30.9 ± 0.3 ^b^	345.9 ± 3.0 ^b^	40.0 ± 2.4 ^b^
Hc-3	23.5 ± 0.5 ^c^	262.7 ± 5.3 ^c^	29.4 ± 2.7 ^b,c,d^
Hc-4	23.5 ± 0.7 ^c^	363.3 ± 7.4 ^c^	18.0 ± 1.6 ^a^
Hc-5	49.3 ± 0.1 ^d^	551.9 ± 1.5 ^d^	26.7 ± 2.7 ^a,d^
Hc-6	42.4 ± 0.9 ^e^	475.1 ± 10.3 ^e^	39.0 ± 1.9 ^b,c^
Trolox	-	-	81.8 ± 6.1 ^e^

* Mean ± Standard deviation. Values with the same letters in the column do not differ statistically in the Tukey test (*p* > 0.05).
